# Rapidly Building Global Health Security Capacity — Uganda Demonstration Project, 2013

**Published:** 2014-01-31

**Authors:** Jeff N. Borchert, Jordan W. Tappero, Robert Downing, Trevor Shoemaker, Prosper Behumbiize, Jane Aceng, Issa Makumbi, Melissa Dahlke, Bassam Jarrar, Briana Lozano, Sam Kasozi, Mark Austin, Dru Phillippe, Ian D. Watson, Tom J. Evans, Timothy Stotish, Scott F. Dowell, Michael F. Iademarco, Raymond Ransom, Arunmozhi Balajee, Kristin Becknell, Denise Beauvais, Tadesse Wuhib

**Affiliations:** 1 Division of Vector-Borne Diseases, National Center for Emerging and Zoonotic Infectious Diseases, CDC; 2 Division of Global Health Protection, Center for Global Health, CDC; 3 Division of Global HIV/AIDS, National Center for Global HIV/AIDS, National Center for Global Health, CDC; 4 Division of High Consequence Pathogens and Pathology, National Center for Emerging and Zoonotic Infectious Diseases, CDC; 5 Uganda Ministry of Health; 6 African Field Epidemiology Network; 7 Division of Emergency Operations, National Center for Global Health, CDC; 8 US Department of Defense, Defense Threat Reduction Agency; 9 Division of TB Elimination, National Center for HIV/AIDS, Viral Hepatitis, STD, and TB Prevention, CDC

Increasingly, the need to strengthen global capacity to prevent, detect, and respond to public health threats around the globe is being recognized. CDC, in partnership with the World Health Organization (WHO), has committed to building capacity by assisting member states with strengthening their national capacity for integrated disease surveillance and response as required by International Health Regulations (IHR) (1,2). CDC and other U.S. agencies have reinforced their pledge through creation of global health security (GHS) demonstration projects. One such project was conducted during March–September 2013, when the Uganda Ministry of Health (MoH) and CDC implemented upgrades in three areas: 1) strengthening the public health laboratory system by increasing the capacity of diagnostic and specimen referral networks, 2) enhancing the existing communications and information systems for outbreak response (3), and 3) developing a public health emergency operations center (EOC) (Figure 1). The GHS demonstration project outcomes included development of an outbreak response module that allowed reporting of suspected cases of illness caused by priority pathogens via short messaging service (SMS; i.e., text messaging) to the Uganda District Health Information System (DHIS-2) and expansion of the biologic specimen transport and laboratory reporting system supported by the President’s Emergency Plan for AIDS Relief (PEPFAR). Other enhancements included strengthening laboratory management, establishing and equipping the EOC, and evaluating these enhancements during an outbreak exercise. In 6 months, the project demonstrated that targeted enhancements resulted in substantial improvements to the ability of Uganda’s public health system to detect and respond to health threats.

MoH chose three priority pathogens (i.e., those in Uganda most likely to contribute to public health emergencies of international concern) as indicators to assess enhancements made through implementation of the project: 1) multidrug-resistant (including extensively drug-resistant) *Mycobacterium tuberculosis*, 2) *Vibrio cholerae,* and 3) Ebola virus, a cause of viral hemorrhagic fever. Of Uganda’s 112 districts, 17 were selected as demonstration project districts (Figure 2) based on the presence of a functional PEPFAR-supported early infant diagnosis (4) specimen transportation network (in which blood spot specimens obtained by heel stick are transported to the capital), availability of equipment for detecting rifampin-resistant *M. tuberculosis* (5), an established viral hemorrhagic fever surveillance site, and a reported cholera outbreak in the preceding 3 years. In each demonstration project district, a toll-free telephone number for reporting an event via SMS and access to DHIS-2 for tracking specimen shipping containers at the EOC and at three national reference laboratories* were provided. Training of laboratory staff members, National District Surveillance Officers, District Laboratory Focal Persons and early infant diagnosis Hub Coordinators was provided for DHIS-2, SMS reporting, sample preparation, packaging, shipping, biosafety, and biosecurity.

In the 17 demonstration project districts, an assessment was conducted in 16 laboratories (seven regional referral hospitals, six general hospitals, and three higher-level health centers†), using a modified WHO laboratory assessment tool (6) that measured differences in laboratory functionality and performance at initiation and completion of the project. Targeted training and mentorship were performed, focusing on safe packaging and transport of specimens using motorcycles and the national postal service for delivery to the relevant national reference laboratory. Rapid diagnostic test kits for toxigenic *Vibrio cholerae* were stocked at district hospitals.

DHIS-2 is an online, open-source, communications system approved by MoH for reporting national health data. The system was enhanced to enable real-time monitoring of suspected-case alerts and response by integrating data sources from the laboratory, transportation, and communication networks with EOC electronic dashboards. New SMS modules were created to allow tracking of specimens. Space was rented adjacent to MoH headquarters to establish a functional EOC with the capacity to receive, evaluate, and distribute information, and to serve as the center of communication and coordination of response operations. The EOC facility was equipped with communications and information technology equipment and staffed with four full-time workers. Manuals and standard operating procedures pertaining to all operational activities were developed.

After 6 months of project implementation, a series of interrelated drills to assess improvements was designed and performed in partnership with the U.S. Department of Defense’s Defense Threat Reduction Agency. The drills measured improvements to laboratory, information, and management systems to effectively prepare for, confirm, notify, and respond to public health emergencies of international concern (1) and included evaluating 1) district, regional, and national level laboratory capabilities for packaging, shipping, receiving, and testing of specimens, as well as reporting of test results within 48 hours of collection; 2) public health information systems across district and national levels, including information flow, data analysis, reporting, and documentation of operational decisions; and 3) public health emergency coordination capabilities at the EOC.

A comprehensive MoH-led plan detailing activities in the three focus areas was first developed. Laboratory upgrades included development of a cold-chain system for specimen transport via the early infant diagnosis transport network, development of testing algorithms for the three priority pathogens, and distribution of standard operating procedures, case definitions, and posters. Although district laboratories reported budget limitations, improvements were observed in all 10 elements of the modified laboratory assessment. At baseline assessment, organizational/management, biorisk/biosafety, and public health function scored the lowest (20%–36%). After this phase of the project, six of the 16 laboratories had improved scores for organizational/management, 10 had improved for documentation, and three had improved for biorisk/biosafety. The greatest progress was observed in public health function (i.e., disease recognition, communication, and specimen transport), where 14 of the 16 laboratories improved, scoring 34%–55%. The 16 laboratories averaged 14% improvement over their original scores and improved in all categories.

The informatics capacity at seven regional referral hospitals was evaluated to assess each laboratory’s ability to process samples and data. Customized modules for each priority pathogen were built into DHIS-2, allowing bidirectional flow of information, SMS notification, and feedback upon sample registration, shipping, receipt, testing, and reporting. A system for tracking alerts, updates, and responses was created to allow EOC monitoring of suspected cases and specimens through an interactive dashboard. Access to the DHIS-2 system was customized, allowing different levels of access for users on a need-to-know basis. New servers also were installed at MoH and offsite.

The drill was successful in evaluating the three focus areas, particularly the laboratory and information systems. Noted successes included proper handling, packaging, and reporting of specimens by district staff members, delivery of samples to national reference laboratories within 24 hours, and use of the suspected case response modules in DHIS-2. This drill provided a baseline to evaluate future enhancements in Uganda’s GHS activities.

## Editorial Note

CDC provided technical support to MoH to increase GHS capacity for preventing, detecting, and responding to public health threats in Uganda. Learning from this experience, CDC is now collaborating with other parts of the U.S. government and national and international health agencies to determine the most efficient and sustainable approach to enhance capacity building in three health-system areas: detection of health threats through laboratory and other systems, coordination of information and response including through EOCs, and prevention of avoidable health threats. Realizing these areas are interconnected, a holistic approach was taken to enhance the specimen referral, testing, and informatics networks to improve case identification, notification, confirmation, and response to disease outbreaks. This model could be replicated in countries with similar health systems.

All activities in support of MoH must be in accordance with and built upon existing policy, infrastructure, technical capacity, workforce, and health initiatives to enhance established systems, including integrated disease surveillance and response programs. Uganda MoH recently revised its integrated disease surveillance and response plan (7), which is the foundation of IHR implementation and focuses on strengthening the National Surveillance System, an essential component for early detection and initiation of timely public health response for epidemic-prone diseases and other conditions on the National Priority List (7). To date, 80% of IHR signatories have not met their 2012 objectives, including Uganda (8). This project assisted MoH in achieving compliance for at least six identified activities measuring IHR competence.

WHO member states understand the importance of strengthening GHS activities through sustainable approaches that are country-led and owned. CDC’s support for GHS capacity building will work synergistically with established and expandable disease surveillance and response activities, including animal sector initiatives (e.g., the U.S. Agency for International Development’s Emerging Pandemic Threats Program). Additionally, it is vital to coordinate and collaborate with U.S. government, regional, and global partners conducting work on similar health priorities in Uganda to reduce duplication and reinforce the U.S. government commitment to strengthen GHS and promote sustainable IHR compliance.

What is already known on this topic?

Security against epidemic disease threats for all countries is dependent on their capacity to prevent, detect, and respond to outbreaks as early and effectively as possible. However, 80% of International Health Regulations signatories have not met their 2012 objectives, including Uganda. CDC has committed to assist countries with national surveillance and response activities to prevent, detect, and respond to public health threats.

What is added by this report?

This report describes rapid global health security enhancements in Uganda targeting three areas: laboratory systems, information systems, and coordination of information through emergency operations centers. These enhancements resulted in substantial improvements in the ability of Uganda’s public health system to detect and respond to health threats in 6 months.

What are the implications for public health practice?

This report provides a potential model for U.S. government collaborative efforts in building international global health security capacity in other countries.

Since the project completion, the DHIS-2 system and specimen transportation network has been used a number of times to report suspected cases of infection with priority pathogens and transport samples from remote locations. Analysis of samples has led to confirmation of cases of infection with West Nile virus, Zika virus, Crimean-Congo hemorrhagic fever virus, hepatitis E virus, *Neisseria meningitidis*, and multidrug-resistant (including extensively drug-resistant) *M. tuberculosis*. Additionally, MoH activated the EOC twice more in 2013. The first was a mass gathering solar eclipse event in northern Uganda, November 3–5, attended by thousands of Ugandans, tourists, and political dignitaries. EOC measures included sensitizing local health and security staff, prepositioning cholera rapid diagnostic tests, hygiene messaging to visitors, and frequent communication between the EOC, field staff members, and senior MoH personnel. The second activation was to support international airport screening for illness consistent with Middle East respiratory syndrome coronavirus infection among persons returning from the Hajj pilgrimage, October 20–25. Uganda currently is expanding the communications and specimen referral network countrywide.

For all countries, security against epidemic disease is dependent on the capacity to prevent, detect, and respond to outbreaks as early and effectively as possible. The Uganda GHS project was able to record considerable systems improvements that might serve as a model for GHS acceleration in other countries.

## Figures and Tables

**FIGURE 1:**
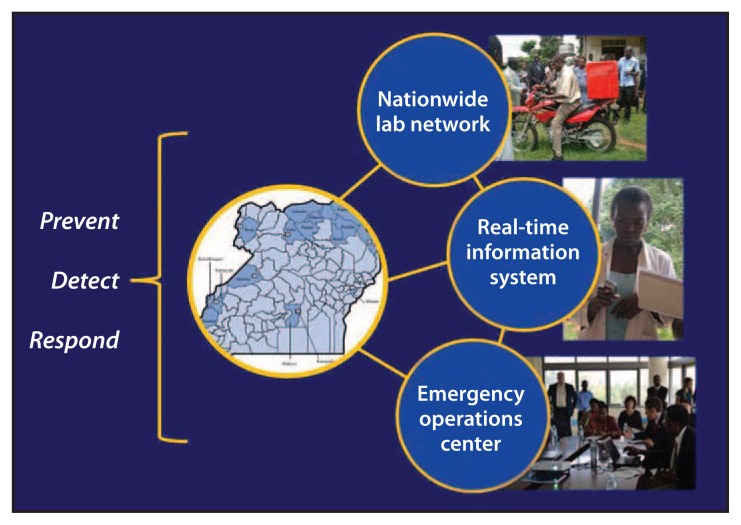
Key upgrades from a global health security demonstration project — Uganda, March–September 2013

**FIGURE 2:**
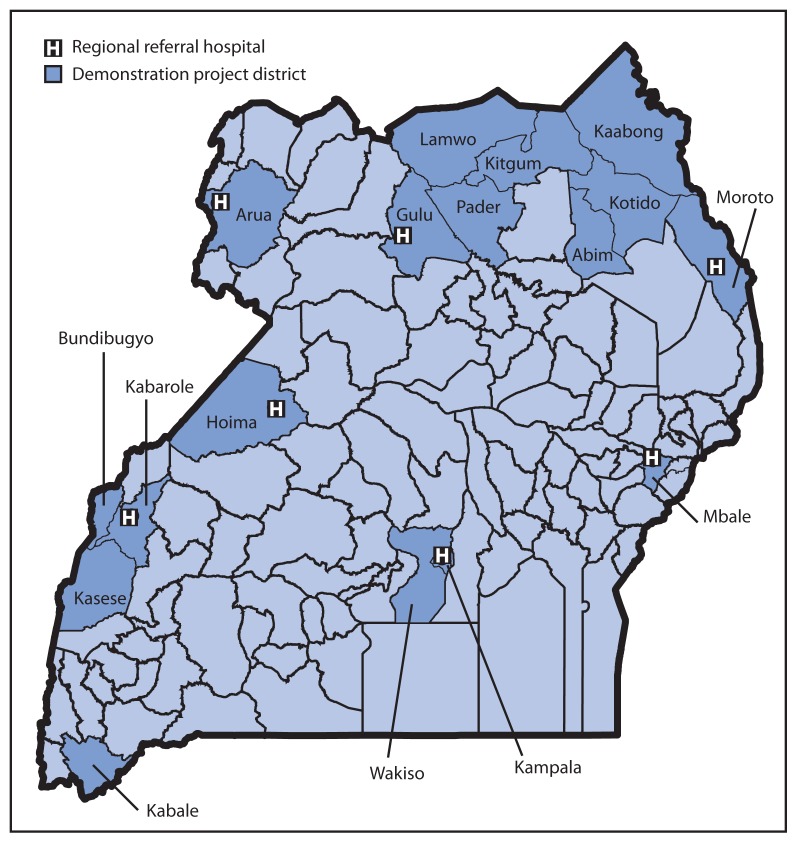
Location of the 17 selected demonstration project districts and seven regional referral hospitals that participated in a global health security demonstration project — Uganda, March–September 2013

## Footnotes

* Central Public Health Laboratory, Uganda Virus Research Institute, and National Tuberculosis Reference Laboratory.

† Level IV clinics, the largest government clinics in counties serving populations of approximately 100,000.
